# Synthesis and crystal structures of multifunctional tosylates as basis for star-shaped poly(2-ethyl-2-oxazoline)s

**DOI:** 10.3762/bjoc.6.96

**Published:** 2010-09-09

**Authors:** Richard Hoogenboom, Martin W M Fijten, Guido Kickelbick, Ulrich S Schubert

**Affiliations:** 1 Laboratory of Macromolecular Chemistry and Nanoscience, Eindhoven University of Technology, Den Dolech 2, 5612AZ Eindhoven, The Netherlands; 2 Supramolecular Chemistry group, Department of Organic Chemistry, Ghent University, Krijgslaan 281 S4, 9000 Ghent, Belgium; 3 Inorganic Solid State Chemistry, Saarland University, Am Markt Zeile 3, 66125 Saarbrücken, Germany; 4 Laboratory of Organic and Macromolecular Chemistry, Friedrich-Schiller-University Jena, Humboldtstrasse 10, 07743 Jena, Germany

**Keywords:** cationic polymerization, crystal structure, living polymerization, star-polymer, tosylate

## Abstract

The synthesis of well-defined polymer architectures is of major importance for the development of complex functional materials. In this contribution, we discuss the synthesis of a range of multifunctional star-shaped tosylates as potential initiators for the living cationic ring-opening polymerization (CROP) of 2-oxazolines resulting in star-shaped polymers. The synthesis of the tosylates was performed by esterification of the corresponding alcohols with tosyl chloride. Recrystallization of these tosylate compounds afforded single crystals, and the X-ray crystal structures of di-, tetra- and hexa-tosylates are reported. The use of tetra- and hexa-tosylates, based on (di)pentaerythritol as initiators for the CROP of 2-ethyl-2-oxazoline, resulted in very slow initiation and ill-defined polymers, which is most likely caused by steric hindrance in these initiators. As a consequence, a porphyrin-cored tetra-tosylate initiator was prepared, which yielded a well-defined star-shaped poly(2-ethyl-2-oxazoline) by CROP as demonstrated by SEC with RI, UV and diode-array detectors, as well as by ^1^H NMR spectroscopy.

## Introduction

Nowadays, well-defined polymer structures are of major importance for the development of ever more sophisticated and complex materials, e.g., applications in drug delivery or as adaptive materials. Star-shaped polymers are especially interesting since their properties are distinctly different from their linear analogues with regard to, e.g., number of functional end-groups, hydrodynamic volume and thermal properties [1–2].

**Figure 1:**
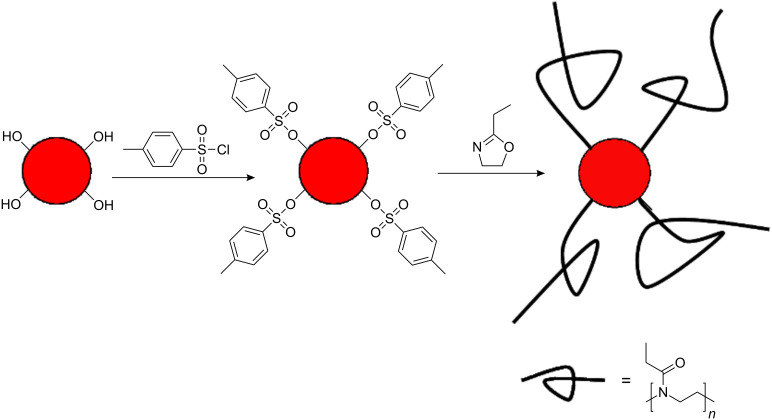
Schematic representation of the investigated strategy for the synthesis of star-shaped poly(2-ethyl-2-oxazoline)s based on multi-tosylate initiators.

Poly(2-oxazoline)s represent a class of versatile polymer structures that can be prepared by the (CROP) of 2-substituted-2-oxazoline monomers (see the bottom right corner of Figure 1 for the structure of poly(2-ethyl-2-oxazoline), as an example of the polymer structure) [3–4]. The versatility of this class of polymers comes from the living nature of the polymerization, allowing control over the length of the polymer with a narrow molar mass distribution and allowing the introduction of specific end-groups by initiation and termination [4]. Moreover, variation of the 2-substituent of the 2-oxazoline results in a variety of the amidic side chains of the poly(2-oxazoline)s [5–6], which strongly influences the properties of the resulting polymers: ranging from hydrophilic to hydrophobic; and from hard materials, with a high glass transition temperature via crystalline and chiral polymers [7], to soft materials, with a very low glass transition temperature [8–9].

The synthesis of star-shaped poly(2-oxazoline)s has been reported using a range of multifunctional electrophilic halide initiators, such as tetrakis(bromomethyl)ethylene, yielding 4-armed star-shaped polymers [10], as well as other multi-halide initiators based on, e.g., cyclotriphosphazine [11], silesquioxane [12], porphyrin [13–14] and bipyridine metal complex [15–16] cores. More recently, Jordan and coworkers reported the use of multi-triflate initiators for the preparation of well-defined star-shaped poly(2-methyl-2-oxazoline)s [17]. However, these multi-halide as well as multi-triflate initiators are not easily prepared and are not stable upon storage, in particular in the presence of air. Therefore, we recently reported a post-modification route for the synthesis of star-shaped poly(2-ethyl-2-oxazoline) by coupling of an acetylene-functionalized poly(2-ethyl-2-oxazoline) to a *heptakis*-azido functionalized *β*-cyclodextrin [18]. However, this method required chromatographic separation of the star-shaped polymer from the acetylene-precursor polymer.

To overcome the limitations of multi-halide, multi-triflate initiators and post-modification methods, we investigated the use of multi-tosylate initiators for the CROP of 2-oxazolines as depicted in Figure 1. The advantages of multi-tosylate initiators are their straightforward syntheses starting from commercially available alcohols, their easy purification based on their high tendency to crystallize [19–20] and their stability under ambient conditions. In this contribution, we report the synthesis and crystal structures of various multi-tosylate initiators as well as their use for the initiation of the CROP of 2-ethyl-2-oxazoline for the formation of star-shaped poly(2-oxazoline)s.

## Results and Discussion

### Multi-tosylate preparation

**Scheme 1:**
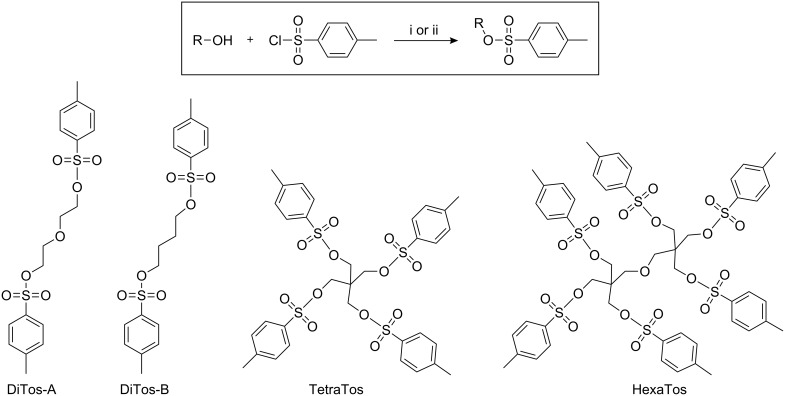
General reaction scheme for the preparation of multi-tosylates from multifunctional alcohols (top) and schematic representation of the structures of the investigated multi-tosylates (bottom). i: triethylamine in dichloromethane; ii: pyridine.

The preparation of tosylates from alcohols is a straightforward synthetic procedure using *p*-toluenesulfonic acid chloride (tosyl chloride) in the presence of a base. Commonly applied procedures are performed in dichloromethane using triethylamine as base or using pyridine both as solvent and base (Scheme 1, top). The synthesis of diethyleneglycol ditosylate (DiTos-A; Scheme 1) was not required since this compound is commercially available. Recrystallization of this compound from ethanol resulted in single crystals suitable for X-ray analysis. Butane ditosylate (DiTos-B; Scheme 1) was synthesized from 1,4-butanediol and an excess of tosyl chloride using dichloromethane as solvent and triethylamine as base. After 24 h stirring at ambient temperature, ethanolamine was added to this reaction mixture to react with the excess of tosyl chloride resulting in the water-soluble 1-hydroxy-2-ethyl tosylamide, which could be removed by washing with 3 N hydrochloric acid and brine. Final purification was performed by recrystallization from ethanol.

The synthesis of the tetra-tosylate (TetraTos; Scheme 1) and hexa-tosylate (HexaTos; Scheme 1) compounds was based on tosylation of pentaerythritol and dipentaerythritol, respectively, which are both insoluble in dichloromethane. Therefore, the solvent was changed to pyridine, which also acts as a base. After the reaction, the mixture was poured into acidified water resulting in the precipitation of the product that could be purified by recrystallization from a mixture of ethanol and acetone.

**Figure 2:**
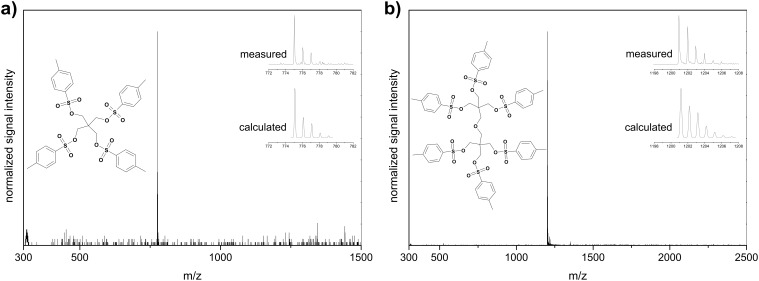
MALDI-TOF MS spectra of TetraTos a) and HexaTos b) Matrix: dithranol.

Besides the common characterization techniques to prove the purity of the compounds, i.e. ^1^H and ^13^C NMR spectroscopy and elemental analysis, the chemical structures of the TetraTos and HexaTos were verified by MALDI-TOF MS, revealing only the desired mass peak corresponding to full tosylation (Figure 2). The absence of residual hydroxyl groups is of major importance for the use of these multi-tosylates as initiators for the CROP of 2-oxazolines since they lead to side reactions with the cationic oxazolinium propagating species.

### Multi-tosylate crystal structures

**Figure 3:**
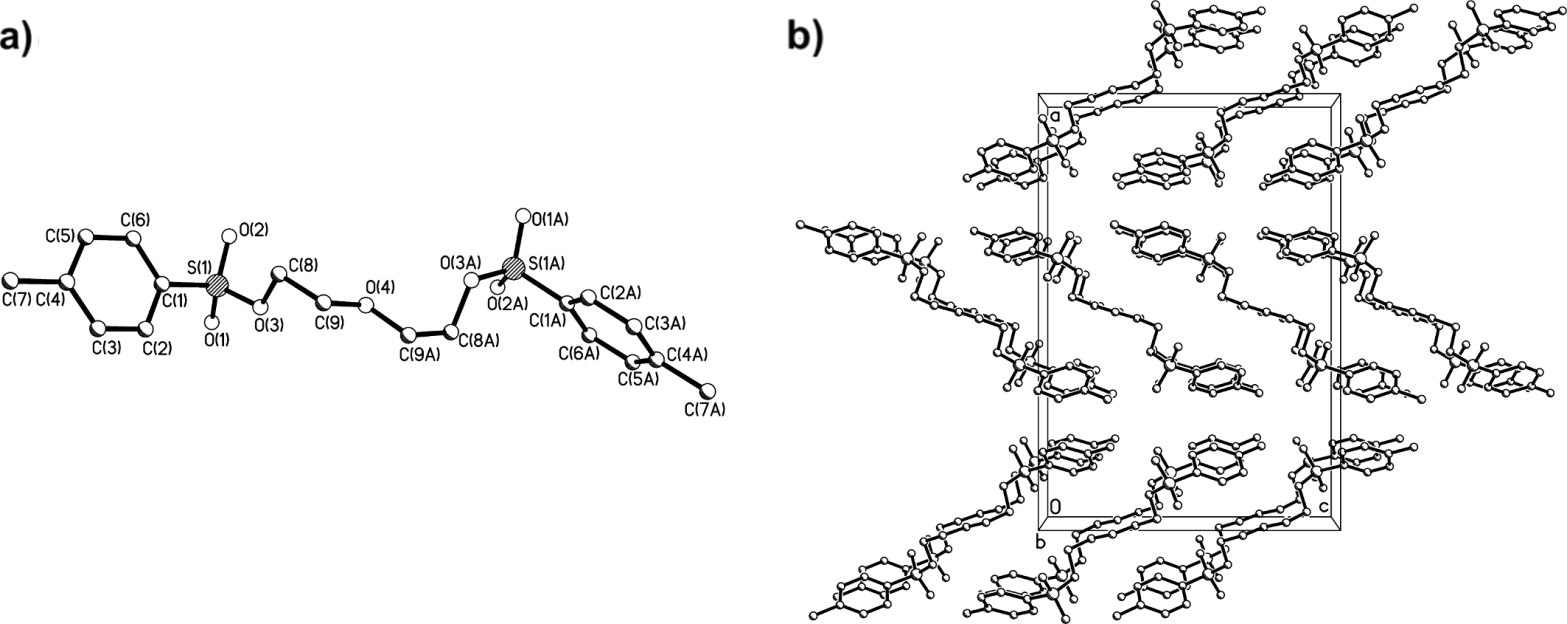
Molecular structure a) and packing diagram b) of the structure of diethyleneglyclol ditosylate (DiTos-A).

**Figure 4:**
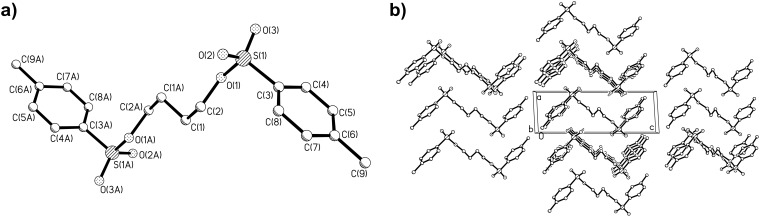
Molecular structure a) and packing diagram b) of the structure of 1,4-butanediol ditosylate (DiTos-B).

**Figure 5:**
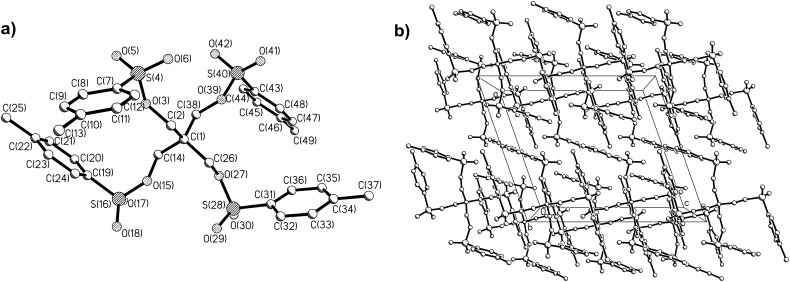
Molecular structure a) and packing diagram b) of the structure of penthaerythritol tetra-tosylate (TetraTos).

**Figure 6:**
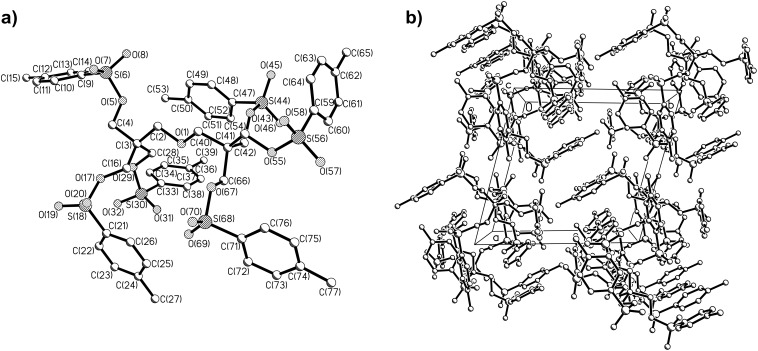
Molecular structure a) and packing diagram b) of the structure of dipenthaerythritol tetra-tosylate (HexaTos).

Recrystallization of the prepared multi-tosylates from ethanol or ethanol–acetone mixtures directly gave single crystals suitable for X-ray analysis as we previously also observed for a tosylate adduct of *2,2’:6’,2’’*-terpyridine [20]. The obtained molecular structures and the packing diagrams for DiTos-A, DiTos-B, TetraTos and HexaTos are displayed in Figures 3–6, respectively. The crystallographic data, selected bond lengths and angles for the crystal structures can be found in the supporting information. All structures show the expected bond length and angles. The packing diagrams reveal space filling packing without any *π*-stacking between the molecules.

### Polymerizations

**Figure 7:**
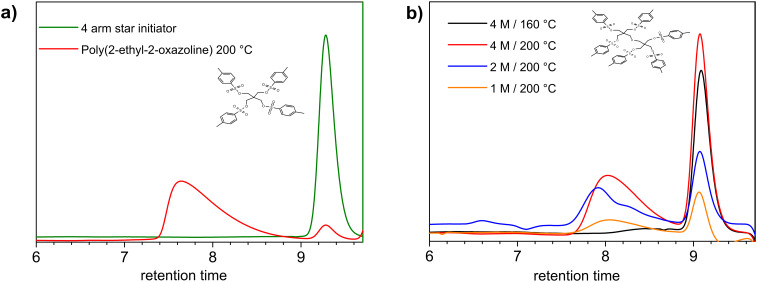
SEC traces obtained for the polymerization of 2-ethyl-2-oxazoline initiated with TetraTos a) and HexaTos b). The large signals at 9 to 9.5 min retention time correspond to the multi-tosylate initiators.

Since the goal of this research was the development of multi-tosylate initiators for the preparation of well-defined star-shaped poly(2-oxazoline)s, TetraTos and HexaTos were utilized for the CROP of 2-ethyl-2-oxazoline under microwave irradiation. Using the optimal polymerization conditions that were previously determined for 2-ethyl-2-oxazoline with methyl tosylate as initiator, i.e. 4 M monomer concentration in acetonitrile, at 140 °C and 10 min for a monomer to initiator ratio of 60 [21–22]; no polymerization was observed at all when using TetraTos or HexaTos as initiators. By contrast, when the polymerization with TetraTos was performed at a further elevated temperature of 200 °C, the formation of polymer was observed by SEC (Figure 7a). However, the resulting polymer had a broad molar mass distribution with tailing at the low molar mass side. In addition, residual tosylate initiator was still observed after heating to 200 °C for 10 min. These results clearly demonstrate that initiation with TetraTos is very slow, which has also been observed for the polymerization with, e.g. 1-butyne tosylate, due to the decreased electrophilicity of the initiator when compared to methyl tosylate [18]. However, the rate of initiation is further decreased for TetraTos compared to 1-butyne tosylate, which is most likely due to steric hindrance in TetraTos resulting in a decreased accessibility of the initiating groups. Similar disappointing polymerization results were obtained with HexaTos as initiator as depicted in Figure 7b. In fact, even less of the HexaTos was consumed after 10 min heating to 200 °C at 4 M monomer concentration as a result of the further increased steric hindrance. Variation of temperature or concentration did not improve the polymerization results. Therefore, it can be concluded that these pentaerythritol based multi-tosylate initiators are not suitable for the CROP of 2-oxazolines, which is in sharp contrast with the rather similar pluritriflate initiators reported by Jordan [17]. This difference is most likely related to both the smaller size and the higher reactivity of the triflate groups compared to the tosylates.

**Scheme 2:**
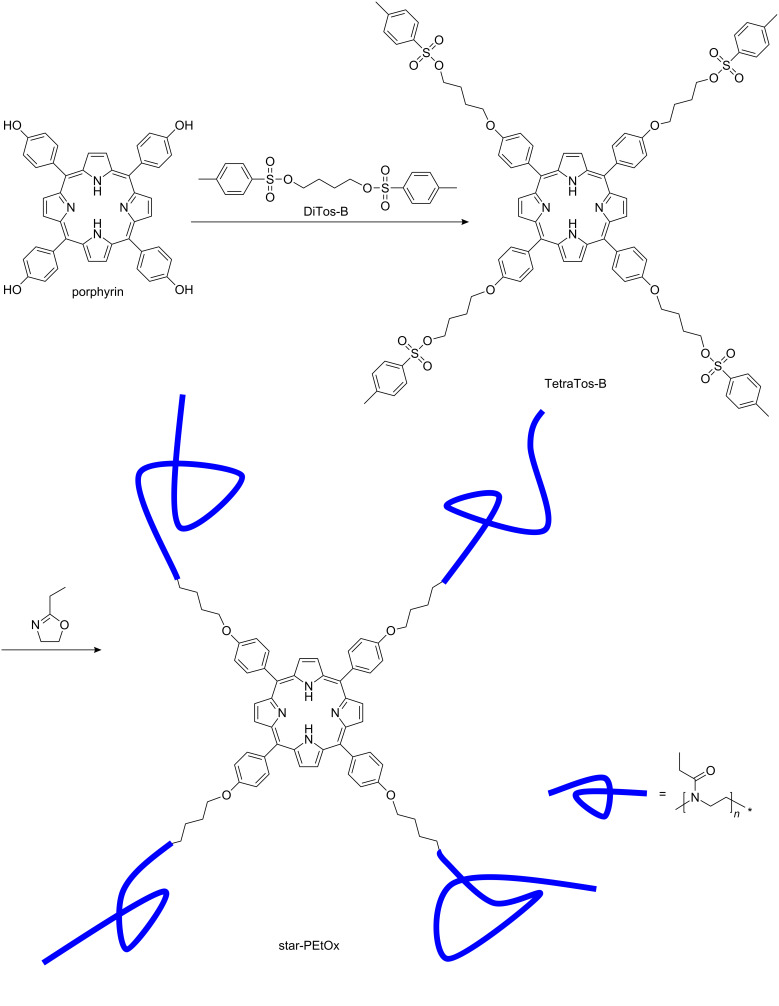
Schematic representation of the synthesis of a porphyrin initiated four-armed star-pEtOx starting from *tetrakis*(hydroxyphenyl)porphyrin (**porphyrin**).

**Figure 8:**
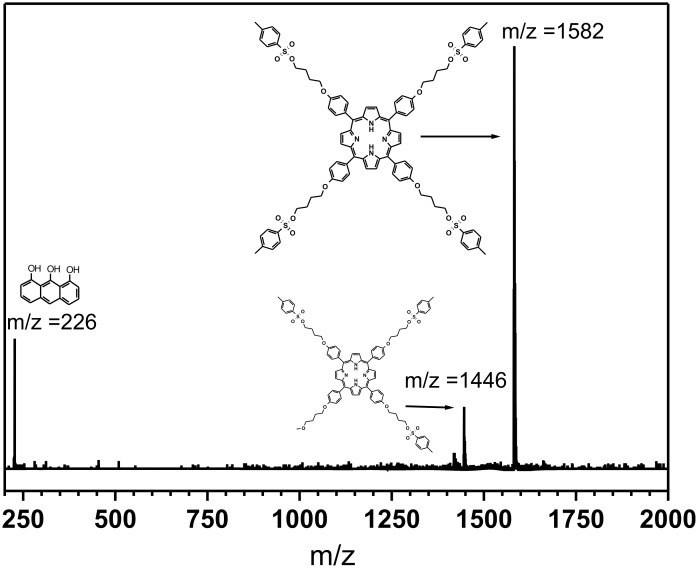
MALDI-TOF MS spectrum of the tetra-tosylate-porphyrin (TetraTos-B). Matrix: dithranol.

To circumvent the poor initiation efficiency with the multi-tosylate pentaerythritol derivatives, a tetra-tosylated porphyrin (TetraTos-B) was designed in which the rigid porphyrin keeps the tosylate groups far apart. Scheme 2 depicts the schematic path that was followed to synthesize TetraTos-B, and subsequently, the four-armed star pEtOx. *Tetrakis*(hydroxyphenyl)porphyrin (porphyrin) was used to synthesize the rigid star-shaped TetraTos-B initiator by reaction with a 20-fold excess of 1,4-butane ditosylate (DiTos-B), followed by chromatographic purification. This is not a straightforward synthesis compared to the previously discussed multi-tosylates, partly counteracting the advantages of multi-tosylate initiators compared to multi-halides and multi-triflates. Figure 8 depicts the MALDI-TOF MS spectrum of the porphyrin initiator TetraTos-B. The formation of TetraTos-B (mass 1582) and a minor fraction, with only three tosylate groups and one methoxybutoxy group (mass 1446), next to the utilized matrix dithranol (mass 226), are clearly evident in the spectrum (Figure 8). This latter methoxylated side chain might have been present in the DiTOs-B. Importantly, no hydroxyl groups remained that might cause side reactions during the polymerizations.

**Figure 9:**
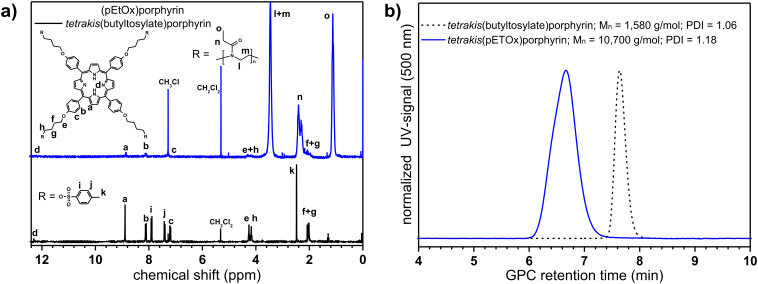
a) ^1^H NMR spectra (in CDCl_3_) of the porphyrin initiator TetraTos-B (bottom) and star-pEtOx (top). b) SEC traces of TetraTos-B and star-PEtOx (in CHCl_3_:NEt_3_:2-PrOH; UV-detector at 500 nm).

Subsequently, this tetrafunctional initiator was applied for the microwave-assisted cationic ring-opening polymerization of EtOx. The polymerization of EtOx with TetraTos-B as initiator was performed with 2 M monomer concentration in CH_3_CN at 140 °C under microwave irradiation with a [M]/[I] ratio of 200, corresponding to 50 monomer units per tosylate group. After 20 min polymerization time, the formation of the polymer was observed and the initiator was completely consumed, which is in clear contrast with TetraTos-A, indicating that indeed decreasing the steric hindrance significantly improves the initiation efficiency of the polymerization. The resulting porphyrin centered star-shaped poly(2-ethyl-2-oxazoline) star-PEtOx was purified by preparative SEC to remove unreacted monomer since precipitation was unsuccessful due to the small scale of the polymerization. Figure 9a depicts the ^1^H NMR spectra of the tosylate-porphyrin TetraTos-B (bottom) and the star-PEtOx (top). The porphyrin signals are still present in the star-PEtOx spectrum, indicating that indeed a four-armed pEtOx with a porphyrin core was synthesized. Integration of both the polymer backbone signals (l and m) and the porphyrin signals (a and b) revealed that 188 EtOx units were incorporated into the polymer, corresponding to 47 monomers per arm, which is close to the theoretical number of 50.

**Figure 10:**
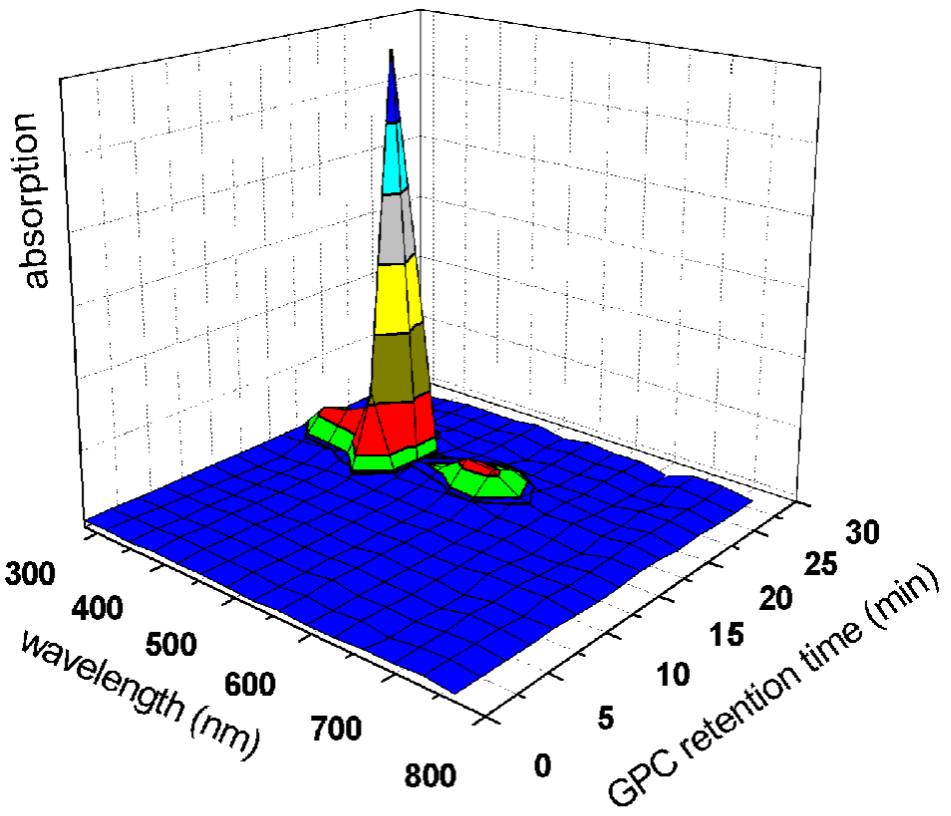
SEC spectrum obtained for star-pEtOx utilizing a photodiode-array detector (eluent: DMF containing 5 mM NH_4_PF_6_).

SEC characterization of the TetraTos-B resulted in a negative signal in the RI-detector, indicating that the porphyrin has a lower RI than the eluent, and a positive signal in the UV-detector at 500 nm, where the porphyrin has a strong UV-absorption (Figure 9b). The *M*_n_ was calculated to be 1,580 g/mol with a polydispersity index (PDI) of 1.06 (against polystyrene standards). This PDI value results from diffusion of the organic compound in the column since it is almost monodisperse (see MALDI in Figure 8). The star-PEtOx could not be characterized with the RI-detector due to the combination of a positive signal of the polymer and a negative signal of the porphyrin. However, detection with the UV-detector at 500 nm revealed a relatively narrow molar mass distribution (Figure 9b), proving that the porphyrin is incorporated into the polymer. The SEC analysis with the UV-detector yielded a *M*_n_ of 10,700 g/mol and a PDI of 1.18 based on linear poly(ethylene glycol) standards. The *M*_n_ is lower than the theoretical molar mass (~21,000 g/mol) due to calibration with linear standards with a different molecular structure. The hydrodynamic volume, that determines the retention time, will be very different for a star-shaped polymer when compared to a linear polymer. Nonetheless, the narrow molar mass distribution indicates that the star-PEtOx was synthesized in a controlled manner. Critical examination of the SEC trace does show a slight shoulder at shorter retention times, indicating the occurrence of minor side reaction leading to star-star coupling. In addition, the specific porphyrin absorption spectrum could be detected in the entire molar mass distribution using SEC with a photodiode-array detector, indicating that the porphyrin is indeed incorporated in all polymer chains (Figure 10).

## Conclusion

The synthesis of various multi-tosylates was successfully performed by esterification of the corresponding alcohols with tosyl chloride. The tosylation of (di)pentaerythritols was only successful using pyridine as solvent due to the limited solubility of the respective educts in dichloromethane. Recrystallization of these tosylate compounds yielded single crystals, and the X-ray crystal structures of di-, tetra- and hexa-tosylates were centrosymmetric with ideal space filling packing. The use of tetra- and hexa-tosylates, based on (di)pentaerythritol as initiators for the living cationic ring-opening polymerization (CROP) of 2-ethyl-2-oxazoline, resulted in very slow initiation and ill-defined polymers, which is most likely due to the steric hindrance of the multiple tosylate groups in these initiators. Therefore, a porphyrin-cored tetra-tosylate initiator with significantly reduced steric hindrance was successfully prepared by reaction of 1,4-butane-ditosylate with 5,10,15,20-*tetrakis*(4-hydroxyphenyl)porphyrin. Utilization of this star-shaped initiator yielded a well-defined star-shaped poly(2-ethyl-2-oxazoline) by CROP.

## Experimental

### Materials

Solvents were purchased from Biosolve Ltd. Acetonitrile (size 3 Å) was dried over molecular sieves. CH_2_Cl_2_ was distilled over potassium. All other solvents were used without further purification. EtOx (Aldrich) was distilled over barium oxide (BaO) and stored under argon. Methyl tosylate (Aldrich) was distilled over P_2_O_5_ and stored under argon. Diethyleneglycol ditosylate (DiTos-A; Aldrich) was recrystallized from ethanol and 5,10,15,20-*tetrakis*(4-hydroxyphenyl)porphyrin (porphyrin; Aldrich) was used without further purification.

### Instrumentation

Polymerizations were carried out in an Emrys Liberator (Biotage, formerly PersonalChemistry) with capped reaction vials. All microwave polymerizations were performed with temperature control (IR sensor).

NMR spectra were recorded on a Varian AM-400 spectrometer or on a Varian Gemini 300 spectrometer. Chemical shifts are given in ppm relative to TMS or residual solvent signals.

Size exclusion chromatography (SEC) was measured on a Shimadzu system with a SCL-10A system controller, a LC-10AD pump, a RID-6A refractive index detector, a SPD-10A UV detector and a PLgel 5 μm Mixed-D column with chloroform: triethylamine:2-propanol (94:4:2) as eluent and the column oven set to 50 °C (polystyrene calibration). SEC with photodiode-array detector was measured on a Waters system with a 1515 pump, a 2414 refractive index detector and a Waters Styragel HT4 column utilizing DMF containing 5 mM NH_4_PF_6_ at a flow rate of 0.5 mL/min as eluent and the column oven set to 50 °C (PEG calibration).

MALDI-TOF-MS was performed on a Voyager-DE™ PRO Biospectrometry™ Workstation (Applied Biosystems) time-of-flight mass spectrometer using the linear mode for operation (positive ion mode; ionization with a 337 nm pulsed nitrogen laser). Elemental analyses were performed on a EuroEA3000 Series EuroVector Elemental Analyzer for CHNS-O.

X-ray crystal structures were measured by mounting selected crystals on a Bruker-AXS APEX diffractometer with a CCD area detector. Graphite-monochromated Mo-K_α_ radiation (71.073 pm) was used for the measurements. The nominal crystal-to-detector distance was 5.00 cm. A hemisphere of data was collected by a combination of three sets of exposures at 292 K. Each set had a different Φ angle for the crystal, and each exposure took 20 s and in steps of 0.3° in ω. The data were corrected for polarization and Lorentz effects, and an empirical absorption correction (SADABS) was applied [23]. The cell dimensions were refined with all unique reflections. The structures were solved by direct methods (SHELXS97). Refinement was carried out with the full-matrix least-squares method based on *F*^2^ (SHELXL97) [24] with anisotropic thermal parameters for all non-hydrogen atoms. Hydrogen atoms were inserted in calculated positions and refined riding with the corresponding atom.

#### Synthesis of 1,4-butanediol ditosylate (DiTos-B)

To a solution of 1,4-butanediol (4.5 g, 50 mmol) and triethylamine (6.07 g, 60 mmol) in dry CH_2_Cl_2_ (100 mL), a solution of tosyl chloride (23.8 g, 125 mmol) in CH_2_Cl_2_ (100 mL) was added dropwise over 75 min. The resulting solution was stirred for 24 h under argon and subsequently, ethanolamine (6 mL) was added to react with excess tosyl chloride. The resulting mixture was poured into water (200 mL). The aqueous layer was extracted with CH_2_Cl_2_, and the combined organic layers were washed successively with 3 N HCl (2 × 100 mL) and brine (150 mL). After drying with MgSO_4_ and filtration, the solvent was evaporated under reduced pressure. Recrystallization of the product from ethanol yielded the desired 1,4-butanediol ditosylate as white platelets in 58% yield (11,5 g, 28,9 mmol).

^1^H NMR (CDCl_3_): δ 7.74 (d, 8.3 Hz, 4H, *o*-CH), 7.33 (d, 8.3 Hz, 4H, *m*-CH), 3.97 (t, 5.5 Hz, 4H, OCH_2_), 2.43 (s, 6H, CH_3_), 1.68 (t, 5.5 Hz, 4H, OCH_2_CH_2_). ^13^C NMR (CDCl_3_): δ 144.8 (CCH_3_), 132.7 (CS), 129.8 (*m*-C), 127.7 (*o*-C), 69.2 (OCH_2_), 24.9 (OCH_2_CH_2_), 21.5 (CCH_3_).

#### Synthesis of pentaerythritol tetra-tosylate (TetraTos)

Pentaerythritol (1.36 g; 10 mmol) and pyridine (20 mL) were weighed into a round-bottom flask and cooled to 0 °C. Subsequently, solid tosyl chloride (9.5 g; 50 mmol) was added portionwise ensuring that the temperature remained below 5 °C. The resulting solution was stirred overnight, during which time it was allowed to warm slowly to ambient temperature. The formed white-pinkish slurry was poured into 125 mL of a 6M HCl solution yielding a white precipitate that was collected by filtration. This solid was washed with water (2 × 100 mL). Further purification was performed by recrystallization from a mixture of ethanol (100 mL) and acetone (100 mL) yielding 4.7 g (62%) of the desired product as white crystals. Partial evaporation of the acetone (~75 mL) from the filtrate yielded another 1.6 g (22%) of crystals, resulting in a total isolated yield of 84%.

^1^H NMR (CDCl_3_): δ 7.68 (d, 8.2 Hz, 8H, *o*-CH_tos_), 7.36 (d, 8.2 Hz, 8H, *m*-CH_tos_), 3.82 (s, 8H, SOCH_2_), 2.47 (s, 12H, CH_3_). ^13^C NMR (CDCl_3_): δ 145.3, 131.0, 129.8, 127.6, 65.2, 42.9, 21.4. C_33_H_36_O_12_S_4_: calcd. C 52.65, H 4.82, S 17.03; found C 52.87, H 4.89, S 17.21.

#### Synthesis of dipentaerythritol hexa-tosylate (HexaTos)

This compound was prepared in a similar manner as TetraTos using the following amounts: dipentaerythritol (2.5 g; 10 mmol), pyridine (20 mL) and tosyl chloride (14.3 g; 75 mmol). Recrystallization of the crude product from ethanol yielded the desired product as white crystals (~8 g; 68%).

^1^H NMR (CDCl_3_): δ 7.66 (d, 8.2 Hz, 12H, *o*-CH_tos_), 7.35 (d, 8.2 Hz, 12H, *m*-CH_tos_), 3.77 (s, 12H, SOCH_2_), 3.14 (s, 4H, OCH_2_) 2.44 (s, 18H, CH_3_). ^13^C NMR (CDCl_3_): δ 145.4, 131.7, 130.0, 127.8, 67.8, 66.5, 43.6, 21.5. C_52_H_58_O_19_S_6_: calcd. C 52.96, H 4.96, S 16.31; found C 53.30, H 5.10, S 15.97.

#### Synthesis of 5,10,15,20-tetrakis(4-hydroxybutyloxy tosylate)-21H,23H-porphyrin (TetraTos-B)

A mixture of 5,10,15,20-*tetrakis* (4-hydroxyphenyl)porphyrin 1 (170 mg, 0.25 mmol), 1,4-butanediol ditosylate 2 (2 g, 5 mmol) and potassium carbonate (190 mg, 1,37 mmol) in dry CH_3_CN was refluxed for 75 h. After this period, the solvent was evaporated under reduced pressure and the residue was redissolved in CHCl_3_. This solution was washed successively with water (100 mL), saturated sodium hydrogen carbonate solution (100 mL) and brine (100 mL). After drying with MgSO_4_ and filtration, the solvent was removed under reduced pressure. The resulting solid was purified by column chromatography (SiO_2_ with CH_2_Cl_2_) and preparative size exclusion chromatography (biobeads SX-1 in CH_2_Cl_2_) resulting in the title compound 3 (38 mg, 0.024 mmol, 10% yield).

^1^H NMR (CDCl_3_): δ 12.3 (s, 2H, NH), 8.89 (s, 8H, CH_por_), 8.20 (d, 8.5 Hz, 8H, OCCHCH), 7.90 (d, 8.2 Hz, 8H, *o*-CH_tos_), 7.42 (d, 8.2 Hz, 8H, *m*-CH_tos_), 7.21 (d, 8.5 Hz, 8H, OCCH), 4.26 (t, 5.6 Hz, 8H, COCH_2_), 4.15 (t, 7.2 Hz, 8H, SOCH_2_), 2.48 (s, 12H, CH_3_), 2.03 (m, 16H, OCH_2_CH_2_CH_2_). ^13^C NMR (CDCl_3_): δ 158.3, 144.5, 135.3, 134.4, 132.9, 129.6, 127.7, 119.4, 112.3, 70.0, 66.8, 25.6, 25.2, 21.3. C_88_H_86_N_4_O_16_S_4_: calcd. C 66.73, H 5.47, N 3.54, S 8.10; found C 66.22, H 5.38, N 3.67, S 7.74. GPC (CHCl_3_:NEt_3_:2-PrOH = 94:4:2; UV detector at 500 nm): *M*_n_ = 1,580 g/mol; PDI = 1.06. MALDI-TOF-MS: *m/z* [M^+^] 1582, [M^+^-tosyl] 1446.

### General polymerization procedure

The polymerizations of 2-ethyl-2-oxazoline with TetraTos and HexaTos as initiators were performed under microwave irradiation. Before use, the microwave vials were heated to 105 °C, allowed to cool to ambient temperature and filled with argon prior to use. Subsequently, the initiator, monomer and acetonitrile were weighed in so that a 1.0 mL polymerization mixture was obtained in which the ratio of monomer per tosylate group is 25 and the desired monomer concentration is 1, 2 or 4 M. This polymerization mixture was heated by microwaves to the desired temperature for a fixed time (5 min at 140 °C; 2 min at 160 °C; 30 seconds or 10 min at 200 °C). After heating, the polymerization mixtures were investigated by size exclusion chromatography.

#### Microwave synthesis of 5,10,15,20-tetrakis(pEtOx)-21H,23H-porphyrin (star-PEtOx)

A mixture of porphyrin tosylate 3 (7.92 mg, 0.005 mmol) and EtOx (100 mg, 1 mmol) in CH_3_CN (0.4 mL) was heated to 140 °C for 20 min under microwave irradiation. After heating, the solvent and residual monomer were evaporated under vacuum and the resulting residue was purified by preparative size exclusion chromatography (biobeads SX-1 with CH_2_Cl_2_), resulting in 60 mg of polymer 4 (56% yield).

^1^H NMR (CDCl_3_): δ 12.23 (s, 2H, NH), 8.85 (br, 8H, CH_por_), 8.10 (br, 8H, 8H, OCCHCH), 7.23 (br, 8H, OCCH), 4.35–4.17 (br, 16H, COCH_2_ + SOCH_2_), 3.75–3.20 (br, 752H, NCH_2_), 2.57–2.05 (br, 395H, COCH_2_ + OCH_2_CH_2_CH_2_), 1.11–1.09 (br, 574H, CH_3_). GPC (CHCl_3_:NEt_3_:2-PrOH = 94:4:2; UV detector at 500 nm): *M*_n_ = 10,700 g/mol; PDI = 1.18.

## Supporting Information

File 1 Details of the reported crystal structures.
