# Supplementary material for: Synthesis and crystal structures of multifunctional tosylates as basis for star-shaped poly(2-ethyl-2-oxazoline)s
Source: Beilstein J Org Chem. 2010 Sep 9;6:773–83. doi: 10.3762/bjoc.6.96 (PMC2956470; doi:10.3762/bjoc.6.96)
Supplement: File 1 — Details of the reported crystal structures. [file Beilstein_J_Org_Chem-06-773-s001.pdf]

Supporting Information

for

Synthesis and crystal structures of multifunctional tosylates as basis for star-shaped poly(2-ethyl-2-oxazoline)s

Richard Hoogenboom*[1,2], Martin W. M. Fijten[1], Guido Kickelbick[3] and Ulrich S. Schubert*[1,4]

Address: [1]Laboratory of Macromolecular Chemistry and Nanoscience, Eindhoven University of Technology, Den Dolech 2, 5612AZ Eindhoven, The Netherlands; [2]Supramolecular Chemistry group, Department of Organic Chemistry, Ghent University, Krijgslaan 281 S4, 9000 Ghent, Belgium; [3]Inorganic Solid State Chemistry, Saarland University, Am Markt Zeile 3, 66125 Saarbrücken, Germany and [4]Laboratory of Organic and Macromolecular Chemistry, Friedrich-Schiller-University Jena, Humboldtstrasse 10, 07743 Jena, Germany

Email: Richard Hoogenboom - r.hoogenboom@tue.nl; Ulrich S. Schubert - ulrich.schubert@uni-jena.de

* Corresponding authors

Table of Contents

Tables 1 and 2: Crystallographic data of DiTos-A S2–S3

Tables 3 and 4: Crystallographic data of DiTos-B S3–S4

Tables 5 and 6: Crystallographic data of TetraTos S5–S7

Tables 7 and 8: Crystallographic data of HexaTos S8–S13

Table 1: Crystal data and structure refinement for DiTos-A.

Empirical formula	$C_{18}H_{22}O_7S_2$	
Formula weight	414.49	
Temperature	296(2) K	
Wavelength	0.71073 A	
Crystal system, space group	orthorhombic, Pbcn	
Unit cell dimensions	a = 2239.7(2) pm	α = 90°
	b = 569.05(5) pm	β = 90°
	c = 1551.74(13) pm	γ = 90°
Volume [x 10^6 pm^3]	1977.7(3)	
Z, Calculated density [g/cm^3]	4, 1.392	
Absorption coefficient [mm^{-1}]	0.306	
F(000)	872	
Crystal size [mm]	0.78 × 0.24 × 0.23	
Theta range for data collection [°]	2.63 to 28.30	
Limiting indices	¯29 ≤ h ≤ 29	
	¯7 ≤ k ≤ 7	
	¯20 ≤ l ≤ 20	
Reflections collected / unique	18775 / 2455 [R(int) = 0.0387]	
Completeness to θ = 28.30 [%]	99.6	
Max. and min. Transmission	0.9330 and 0.7964	
Refinement method	Full-matrix least-squares on	
Data / restraints / parameters	2455 / 0 / 124	
Goodness-of-fit on F^2	1.160	
Final R indices [I>2σ(I)]	R1 = 0.0695, wR2 = 0.1655	
R indices (all data)	R1 = 0.0831, wR2 = 0.1758	
Largest diff. peak and hole [e·$Å^3$]	0.506 and ¯0.428	

Weight = $1 / [\sigma^2(F_o^2) + (0.0597\ )^2 + 2.56\ P]$ where $P = (\mathrm{Max}\ (F_o^2, 0) + 2\ F_c^2) / 3$

Table 2: Selected bond lengths [pm] and angles [°] for DiTos-A.

S(1)-O(1)	141.9(3)
S(1)-O(2)	142.2(3)
S(1)-O(3)	157.7(2)
S(1)-C(1)	175.4(3)
C(1)-C(2)	138.3(4)
C(1)-C(6)	138.7(4)
C(2)-C(3)	138.0(4)
O(3)-C(8)	145.1(4)
C(3)-C(4)	138.1(4)
O(4)-C(9)#1	140.8(4)
O(4)-C(9)	140.8(4)
C(4)-C(5)	138.9(4)
C(4)-C(7)	151.4(4)
C(5)-C(6)	138.0(4)
O(1)-S(1)-O(2)	119.72(18)
O(1)-S(1)-O(3)	103.97(15)
O(2)-S(1)-O(3)	109.29(14)
O(1)-S(1)-C(1)	109.91(15)

O(2)-S(1)-C(1)	108.93(15)
O(3)-S(1)-C(1)	103.79(13)
C(2)-C(1)-C(6)	121.0(3)
C(2)-C(1)-S(1)	119.6(2)
C(6)-C(1)-S(1)	119.4(2)
C(3)-C(2)-C(1)	119.0(3)
C(8)-O(3)-S(1)	117.2(2)
C(2)-C(3)-C(4)	121.3(3)
C(9)#1-O(4)-C(9)	112.2(4)
C(3)-C(4)-C(5)	118.7(3)
C(3)-C(4)-C(7)	120.8(3)
C(5)-C(4)-C(7)	120.5(3)
C(6)-C(5)-C(4)	121.1(3)
C(5)-C(6)-C(1)	118.9(3)
O(3)-C(8)-C(9)	107.5(3)
O(4)-C(9)-C(8)	109.7(3)

Symmetry transformations used to generate equivalent atoms: #1 −x+1,y, −z+1/2.

Table 3: Crystal data and structure refinement for DiTos-B.

Empirical formula	$C_{18}H_{22}O_6S_2$	
Formula weight	398.49	
Temperature	296(2) K	
Wavelength	0.71073 A	
Crystal system, space group	triclinic, P-1	
Unit cell dimensions	a = 520.47(16) pm	α = 90.241(6)°
	b = 603.05(18) pm	β = 93.091(6)°
	c = 1524.9(5) pm	γ = 94.634(6)°
Volume [x 10^6 pm^3]	476.3(2)	
Z, Calculated density [g/cm^3]	2, 1.389	
Absorption coefficient [mm^{-1}]	0.311	
F(000)	210	
Crystal size [mm]	0.80 × 0.33 × 0.06	
Theta range for data collection [°]	2.68 to 28.25	
Limiting indices	−6 ≤ h ≤ 6	
	−8 ≤ k ≤ 7	
	−20 ≤ l ≤ 20	
Reflections collected / unique	4752 / 2326 [R(int) = 0.1126]	
Completeness to θ = 28.2 [%]	99.4	
Max. and min. Transmission	0.9816 and 0.7892	
Refinement method	Full-matrix least-squares on F^2	
Data / restraints / parameters	2326 / 0 / 119	
Goodness-of-fit on F^2	1.076	
Final R indices [I>2σ(I)]	R1 = 0.0588, wR2 = 0.1509	
R indices (all data)	R1 = 0. 1.0760662, wR2 = 0.1575	
Largest diff. peak and hole [e·$Å^3$]	0.584 and −0.280	

Weight = 1 / [$\sigma^2(F_o^2)$ + (0.0713 P $)^2$ + 0.05] where P = (Max (F_o^2, 0) + 2 F_c^2) / 3

Table 4: Selected bond lengths [pm] and angles [°] for DiTos-B.

S(1)-O(3)	141.98(17)
S(1)-O(2)	142.39(18)
S(1)-O(1)	157.10(13)
S(1)-C(3)	175.63(19)
O(1)-C(2)	146.7(2)
C(1)-C(2)	150.8(3)
C(1)-C(1)#1	151.8(4)
C(3)-C(8)	138.1(3)
C(3)-C(4)	138.6(3)
C(4)-C(5)	137.4(3)
C(5)-C(6)	139.0(4)
C(6)-C(7)	138.4(3)
C(6)-C(9)	150.5(3)
C(7)-C(8)	137.4(3)
O(3)-S(1)-O(2)	120.00(11)
O(3)-S(1)-O(1)	103.90(10)
O(2)-S(1)-O(1)	109.00(9)
O(3)-S(1)-C(3)	109.78(10)
O(2)-S(1)-C(3)	108.65(10)
O(1)-S(1)-C(3)	104.34(8)
C(2)-O(1)-S(1)	117.45(12)
C(2)-C(1)-C(1)#1	113.8(2)
O(1)-C(2)-C(1)	107.26(16)
C(8)-C(3)-C(4)	120.60(19)
C(8)-C(3)-S(1)	119.57(15)
C(4)-C(3)-S(1)	119.82(16)
C(5)-C(4)-C(3)	119.0(2)
C(4)-C(5)-C(6)	121.4(2)
C(7)-C(6)-C(5)	118.3(2)
C(7)-C(6)-C(9)	121.0(2)
C(5)-C(6)-C(9)	120.7(2)
C(8)-C(7)-C(6)	121.3(2)
C(7)-C(8)-C(3)	119.39(19)

Symmetry transformations used to generate equivalent atoms: #1 −x+1, −y+2,-z+1

Table 5: Crystal data and structure refinement for TetraTos.

Empirical formula	$C_{33}H_{36}O_{12}S_4$	
Formula weight	752.86	
Temperature	296(2) K	
Wavelength	0.71073 A	
Crystal system, space group	monoclinic, $P2_1/c$	
Unit cell dimensions	a = 1343.50(5) pm	α = 90.241(6)°
	b = 1826.45(7) pm	β = 109.310(1)°
	c = 1551.87(6) pm	γ = 94.634(6)°
Volume [x 10^6 pm^3]	3593.8(2)	
Z, Calculated density [g/cm^3]	4, 1.391	
Absorption coefficient [mm^{-1}]	0.325	
F(000)	1576	
Crystal size [mm]	0.42 × 0.38 × 0.25	
Theta range for data collection [°]	2.63 to 30.05	
Limiting indices	−18 ≤ h ≤ 17	
	−25 ≤ k ≤ 25	
	−21 ≤ l ≤ 19	
Reflections collected / unique	27550 / 10437 [R(int) = 0.0187]	
Completeness to θ = 30.05 [%]	99.3	
Max. and min. Transmission	0.9232 and 0.8756	
Refinement method	Full-matrix least-squares on F^2	
Data / restraints / parameters	10437 / 0 / 442	
Goodness-of-fit on F^2	1.012	
Final R indices [I>2σ(I)]	R1 = 0.0486, wR2 = 0.1290	
R indices (all data)	R1 = 0.0706, wR2 = 0.1463	
Largest diff. peak and hole [e·$Å^3$]	0.364 and -0.278	

Weight = $1 / [\sigma^2(F_o^2) + (0.0850 P)^2 + 0.25 P]$ where $P = (\text{Max}(F_o^2, 0) + 2 F_c^2) / 3$

Table 6: Selected bond lengths [pm] and angles [°] for TetraTos.

C(1)-C(38)	152.3(2)
C(1)-C(2)	152.5(2)
C(1)-C(26)	153.1(2)
C(1)-C(14)	153.3(2)
C(2)-O(3)	145.30(19)
O(3)-S(4)	157.97(14)
S(4)-O(5)	142.07(14)
S(4)-O(6)	142.13(15)
S(4)-C(7)	174.58(19)
C(7)-C(12)	137.9(3)
C(7)-C(8)	139.0(3)
C(8)-C(9)	136.7(3)
C(9)-C(10)	138.1(3)
C(10)-C(11)	138.2(3)
C(10)-C(13)	151.0(3)
C(11)-C(12)	137.0(3)
C(14)-O(15)	145.00(18)
O(15)-S(16)	157.88(11)

S(16)-O(18)	142.14(13)
S(16)-O(17)	142.49(12)
S(16)-C(19)	174.73(16)
C(19)-C(20)	137.8(2)
C(19)-C(24)	138.7(2)
C(20)-C(21)	138.3(3)
C(21)-C(22)	137.3(3)
C(22)-C(23)	137.7(3)
C(22)-C(25)	151.1(3)
C(23)-C(24)	137.5(3)
C(26)-O(27)	145.51(19)
O(27)-S(28)	157.99(13)
S(28)-O(29)	142.05(14)
S(28)-O(30)	142.25(14)
S(28)-C(31)	174.05(19)
C(31)-C(36)	138.3(2)
C(31)-C(32)	138.5(2)
C(32)-C(33)	136.8(3)
C(33)-C(34)	137.9(3)
C(34)-C(35)	138.1(3)
C(34)-C(37)	151.1(3)
C(35)-C(36)	136.6(3)
C(38)-O(39)	145.21(17)
O(39)-S(40)	158.10(11)
S(40)-O(42)	141.92(14)
S(40)-O(41)	142.16(12)
S(40)-C(43)	175.28(17)
C(43)-C(48)	138.5(3)
C(43)-C(44)	138.5(2)
C(44)-C(45)	138.4(3)
C(45)-C(46)	137.7(3)
C(46)-C(47)	138.1(3)
C(46)-C(49)	150.8(3)
C(47)-C(48)	137.3(3)
C(38)-C(1)-C(2)	111.33(13)
C(38)-C(1)-C(26)	111.26(12)
C(2)-C(1)-C(26)	106.42(12)
C(38)-C(1)-C(14)	105.84(12)
C(2)-C(1)-C(14)	110.99(12)
C(26)-C(1)-C(14)	111.08(13)
O(3)-C(2)-C(1)	107.51(12)
C(2)-O(3)-S(4)	119.26(10)
O(5)-S(4)-O(6)	119.77(10)
O(5)-S(4)-O(3)	103.79(9)
O(6)-S(4)-O(3)	108.14(8)
O(5)-S(4)-C(7)	110.02(9)
O(6)-S(4)-C(7)	109.01(9)
O(3)-S(4)-C(7)	105.03(8)
C(12)-C(7)-C(8)	120.16(18)
C(12)-C(7)-S(4)	120.10(15)
C(8)-C(7)-S(4)	119.73(15)
C(9)-C(8)-C(7)	119.43(19)

C(8)-C(9)-C(10)	121.3(2)
C(9)-C(10)-C(11)	118.3(2)
C(9)-C(10)-C(13)	121.0(2)
C(11)-C(10)-C(13)	120.7(2)
C(12)-C(11)-C(10)	121.5(2)
C(11)-C(12)-C(7)	119.28(19)
O(15)-C(14)-C(1)	106.71(12)
C(14)-O(15)-S(16)	117.92(9)
O(18)-S(16)-O(17)	120.46(8)
O(18)-S(16)-O(15)	103.96(7)
O(17)-S(16)-O(15)	108.72(7)
O(18)-S(16)-C(19)	109.29(8)
O(17)-S(16)-C(19)	109.25(8)
O(15)-S(16)-C(19)	103.81(7)
C(20)-C(19)-C(24)	120.66(15)
C(20)-C(19)-S(16)	120.89(12)
C(24)-C(19)-S(16)	118.44(13)
C(19)-C(20)-C(21)	118.59(16)
C(22)-C(21)-C(20)	121.94(18)
C(21)-C(22)-C(23)	118.22(18)
C(21)-C(22)-C(25)	121.3(2)
C(23)-C(22)-C(25)	120.52(19)
C(24)-C(23)-C(22)	121.59(16)
C(23)-C(24)-C(19)	119.00(16)
O(27)-C(26)-C(1)	106.92(12)
C(26)-O(27)-S(28)	118.59(10)
O(29)-S(28)-O(30)	119.56(10)
O(29)-S(28)-O(27)	108.75(8)
O(30)-S(28)-O(27)	103.55(8)
O(29)-S(28)-C(31)	109.20(9)
O(30)-S(28)-C(31)	109.78(9)
O(27)-S(28)-C(31)	104.92(8)
C(36)-C(31)-C(32)	120.08(18)
C(36)-C(31)-S(28)	119.71(14)
C(32)-C(31)-S(28)	120.16(14)
C(33)-C(32)-C(31)	119.23(18)
C(32)-C(33)-C(34)	121.65(19)
C(33)-C(34)-C(35)	118.1(2)
C(33)-C(34)-C(37)	120.3(2)
C(35)-C(34)-C(37)	121.6(2)
C(36)-C(35)-C(34)	121.6(2)
C(35)-C(36)-C(31)	119.39(19)
O(39)-C(38)-C(1)	107.28(11)
C(38)-O(39)-S(40)	117.41(9)
O(42)-S(40)-O(41)	120.34(9)
O(42)-S(40)-O(39)	109.00(7)
O(41)-S(40)-O(39)	103.77(7)
O(42)-S(40)-C(43)	109.03(8)
O(41)-S(40)-C(43)	109.45(8)
O(39)-S(40)-C(43)	103.96(7)
C(48)-C(43)-C(44)	120.51(18)
C(48)-C(43)-S(40)	119.64(13)

C(44)-C(43)-S(40)	119.85(14)
C(45)-C(44)-C(43)	118.58(19)
C(46)-C(45)-C(44)	121.88(19)
C(45)-C(46)-C(47)	118.1(2)
C(45)-C(46)-C(49)	121.1(2)
C(47)-C(46)-C(49)	120.7(2)
C(48)-C(47)-C(46)	121.6(2)
C(47)-C(48)-C(43)	119.27(18)

Table 7: Crystal data and structure refinement for HexaTos.

Empirical formula	$C_{52}H_{58}O_{19}S_6$	
Formula weight	1179.38	
Temperature	296(2) K	
Wavelength	0.71073 A	
Crystal system, space group	triclinic, P-1	
Unit cell dimensions	a = 1236.49(6)pm	α = 102.551(1)°
	b = 1285.81(6) pm	β = 102.194(1)°
	c = 1977.73(9) pm	γ = 101.605(1)°
Volume [x 10^6 pm^3]	2897.7(2)	
Z, Calculated density [g/cm^3]	2, 1.368	
Absorption coefficient [mm^{-1}]	0.308	
F(000)	1250	
Crystal size [mm]	0.73 × 0.46 × 0.24	
Theta range for data collection [°]	2.36 to 26.37	
Limiting indices	−15 ≤ h ≤ 10	
	−16 ≤ k ≤ 15	
	−24 ≤ l ≤ 24	
Reflections collected / unique	17323 / 11609 [R(int) = 0.0158]	
Completeness to θ = 26.37 [%]	98.0	
Max. and min. Transmission	0.9297 and 0.8063	
Refinement method	Full-matrix least-squares on F^2	
Data / restraints / parameters	11609 / 0 / 712	
Goodness-of-fit on F^2	1.036	
Final R indices [I>2σ(I)]	R1 = 0.0607, wR2 = 0.1736	
R indices (all data)	R1 = 0.0760, wR2 = 0.1912	
Largest diff. peak and hole [e·$Å^3$]	1.605 and −0.553	

Weight = 1 / [$\sigma^2(F_o^2) + (0.1170\ P)^2 + 1.06\ P$]   where P = (Max ($F_o^2$, 0) + 2 F_c^2) / 3

Table 8: Selected bond lengths [pm] and angles [°] for HexaTos.

O(1)-C(2)	141.7(3)
O(1)-C(40)	141.8(3)
C(2)-C(3)	153.0(3)
C(3)-C(28)	151.1(4)
C(3)-C(16)	152.8(3)
C(3)-C(4)	153.3(3)
C(4)-O(5)	140.9(4)
O(5)-S(6)	158.8(2)

S(6)-O(7)	135.7(4)
S(6)-O(8)	143.3(3)
S(6)-C(9)	172.8(3)
C(9)-C(14)	137.4(5)
C(9)-C(10)	139.5(4)
C(10)-C(11)	138.7(4)
C(11)-C(12)	135.6(5)
C(12)-C(13)	137.1(5)
C(12)-C(15)	151.2(5)
C(13)-C(14)	136.7(5)
C(16)-O(17)	144.8(3)
O(17)-S(18)	158.35(17)
S(18)-O(19)	142.2(2)
S(18)-O(20)	142.2(2)
S(18)-C(21)	174.8(3)
C(21)-C(26)	137.8(4)
C(21)-C(22)	138.7(4)
C(22)-C(23)	136.5(5)
C(23)-C(24)	138.4(6)
C(24)-C(25)	139.1(5)
C(24)-C(27)	150.3(5)
C(25)-C(26)	137.5(4)
C(28)-O(29)	144.9(3)
O(29)-S(30)	158.3(2)
S(30)-O(31)	141.8(3)
S(30)-O(32)	141.8(2)
S(30)-C(33)	175.0(3)
C(33)-C(34)	137.4(4)
C(33)-C(38)	138.1(4)
C(34)-C(35)	137.0(4)
C(35)-C(36)	138.4(5)
C(36)-C(37)	137.4(5)
C(36)-C(39)	150.8(4)
C(37)-C(38)	137.2(4)
C(40)-C(41)	153.0(3)
C(41)-C(54)	151.9(3)
C(41)-C(66)	152.2(4)
C(41)-C(42)	153.4(3)
C(42)-O(43)	144.6(3)
O(43)-S(44)	158.6(2)
S(44)-O(45)	141.8(3)
S(44)-O(46)	142.1(3)
S(44)-C(47)	175.3(3)
C(47)-C(48)	137.1(4)
C(47)-C(52)	138.4(4)
C(48)-C(49)	136.7(5)
C(49)-C(50)	137.7(6)
C(50)-C(51)	137.4(6)
C(50)-C(53)	150.9(6)
C(51)-C(52)	138.8(6)
C(54)-O(55)	145.4(3)
O(55)-S(56)	157.72(19)

S(56)-O(57)	141.8(2)
S(56)-O(58)	142.0(2)
S(56)-C(59)	175.0(3)
C(59)-C(64)	137.4(5)
C(59)-C(60)	137.9(5)
C(60)-C(61)	137.2(5)
C(61)-C(62)	136.9(6)
C(62)-C(63)	137.5(6)
C(62)-C(65)	151.3(6)
C(63)-C(64)	137.7(6)
C(66)-O(67)	145.3(3)
O(67)-S(68)	158.0(2)
S(68)-O(69)	141.7(3)
S(68)-O(70)	142.1(3)
S(68)-C(71)	174.6(3)
C(71)-C(76)	136.6(5)
C(71)-C(72)	137.1(5)
C(72)-C(73)	139.5(8)
C(73)-C(74)	136.8(9)
C(74)-C(75)	133.0(9)
C(74)-C(77)	149.7(7)
C(75)-C(76)	139.5(5)
C(1S)-O(1S)#1	105(4)
C(1S)-C(1S)#1	105(4)
C(1S)-C(2S)	113(4)
C(1S)-O(2S)#1	191(4)
C(1S)-C(2S)#1	191(4)
C(2S)-O(1S)#1	191(4)
C(2S)-C(1S)#1	191(4)
C(2)-O(1)-C(40)	111.14(18)
O(1)-C(2)-C(3)	109.38(19)
C(28)-C(3)-C(16)	112.4(2)
C(28)-C(3)-C(2)	108.4(2)
C(16)-C(3)-C(2)	106.87(19)
C(28)-C(3)-C(4)	112.0(2)
C(16)-C(3)-C(4)	109.1(2)
C(2)-C(3)-C(4)	107.8(2)
O(5)-C(4)-C(3)	108.7(2)
C(4)-O(5)-S(6)	119.1(2)
O(7)-S(6)-O(8)	120.59(19)
O(7)-S(6)-O(5)	111.53(16)
O(8)-S(6)-O(5)	99.99(16)
O(7)-S(6)-C(9)	110.34(16)
O(8)-S(6)-C(9)	108.96(16)
O(5)-S(6)-C(9)	103.81(13)
C(14)-C(9)-C(10)	120.3(3)
C(14)-C(9)-S(6)	118.9(2)
C(10)-C(9)-S(6)	120.7(2)
C(11)-C(10)-C(9)	117.8(3)
C(12)-C(11)-C(10)	121.8(3)
C(11)-C(12)-C(13)	119.2(3)
C(11)-C(12)-C(15)	120.7(4)

C(13)-C(12)-C(15)	120.1(4)
C(14)-C(13)-C(12)	121.0(3)
C(13)-C(14)-C(9)	119.8(3)
O(17)-C(16)-C(3)	109.31(19)
C(16)-O(17)-S(18)	117.23(15)
O(19)-S(18)-O(20)	119.66(14)
O(19)-S(18)-O(17)	103.67(12)
O(20)-S(18)-O(17)	108.89(12)
O(19)-S(18)-C(21)	109.95(15)
O(20)-S(18)-C(21)	109.57(14)
O(17)-S(18)-C(21)	103.81(11)
C(26)-C(21)-C(22)	121.0(3)
C(26)-C(21)-S(18)	120.3(2)
C(22)-C(21)-S(18)	118.7(2)
C(23)-C(22)-C(21)	118.8(3)
C(22)-C(23)-C(24)	121.9(3)
C(23)-C(24)-C(25)	118.1(3)
C(23)-C(24)-C(27)	121.3(4)
C(25)-C(24)-C(27)	120.5(4)
C(26)-C(25)-C(24)	121.0(3)
C(25)-C(26)-C(21)	119.2(3)
O(29)-C(28)-C(3)	109.0(2)
C(28)-O(29)-S(30)	120.48(18)
O(31)-S(30)-O(32)	120.87(17)
O(31)-S(30)-O(29)	108.71(14)
O(32)-S(30)-O(29)	103.63(14)
O(31)-S(30)-C(33)	109.02(14)
O(32)-S(30)-C(33)	109.72(13)
O(29)-S(30)-C(33)	103.41(12)
C(34)-C(33)-C(38)	120.3(3)
C(34)-C(33)-S(30)	119.5(2)
C(38)-C(33)-S(30)	120.2(2)
C(35)-C(34)-C(33)	119.5(3)
C(34)-C(35)-C(36)	121.4(3)
C(37)-C(36)-C(35)	118.1(3)
C(37)-C(36)-C(39)	120.9(3)
C(35)-C(36)-C(39)	121.0(3)
C(38)-C(37)-C(36)	121.6(3)
C(37)-C(38)-C(33)	119.2(3)
O(1)-C(40)-C(41)	109.45(19)
C(54)-C(41)-C(66)	111.6(2)
C(54)-C(41)-C(40)	105.25(19)
C(66)-C(41)-C(40)	111.6(2)
C(54)-C(41)-C(42)	111.5(2)
C(66)-C(41)-C(42)	106.0(2)
C(40)-C(41)-C(42)	111.04(19)
O(43)-C(42)-C(41)	107.5(2)
C(42)-O(43)-S(44)	118.18(17)
O(45)-S(44)-O(46)	121.01(19)
O(45)-S(44)-O(43)	103.74(15)
O(46)-S(44)-O(43)	108.95(14)
O(45)-S(44)-C(47)	109.22(16)

O(46)-S(44)-C(47)	109.24(16)
O(43)-S(44)-C(47)	103.12(12)
C(48)-C(47)-C(52)	119.9(3)
C(48)-C(47)-S(44)	119.0(2)
C(52)-C(47)-S(44)	121.0(2)
C(49)-C(48)-C(47)	120.2(3)
C(48)-C(49)-C(50)	121.9(3)
C(51)-C(50)-C(49)	117.1(4)
C(51)-C(50)-C(53)	121.9(4)
C(49)-C(50)-C(53)	121.0(4)
C(50)-C(51)-C(52)	122.6(3)
C(47)-C(52)-C(51)	118.2(3)
O(55)-C(54)-C(41)	108.3(2)
C(54)-O(55)-S(56)	115.43(16)
O(57)-S(56)-O(58)	120.08(15)
O(57)-S(56)-O(55)	104.40(13)
O(58)-S(56)-O(55)	108.62(11)
O(57)-S(56)-C(59)	110.33(14)
O(58)-S(56)-C(59)	109.57(16)
O(55)-S(56)-C(59)	102.21(12)
C(64)-C(59)-C(60)	120.1(3)
C(64)-C(59)-S(56)	121.6(3)
C(60)-C(59)-S(56)	118.3(3)
C(61)-C(60)-C(59)	119.5(4)
C(62)-C(61)-C(60)	121.6(4)
C(61)-C(62)-C(63)	117.8(4)
C(61)-C(62)-C(65)	121.5(5)
C(63)-C(62)-C(65)	120.7(5)
C(62)-C(63)-C(64)	122.1(4)
C(59)-C(64)-C(63)	118.8(4)
O(67)-C(66)-C(41)	107.22(19)
C(66)-O(67)-S(68)	117.68(16)
O(69)-S(68)-O(70)	121.0(2)
O(69)-S(68)-O(67)	108.88(15)
O(70)-S(68)-O(67)	103.69(14)
O(69)-S(68)-C(71)	108.2(2)
O(70)-S(68)-C(71)	109.72(16)
O(67)-S(68)-C(71)	103.96(13)
C(76)-C(71)-C(72)	119.7(4)
C(76)-C(71)-S(68)	119.3(3)
C(72)-C(71)-S(68)	120.9(3)
C(71)-C(72)-C(73)	118.6(5)
C(74)-C(73)-C(72)	122.2(5)
C(75)-C(74)-C(73)	117.6(5)
C(75)-C(74)-C(77)	122.4(8)
C(73)-C(74)-C(77)	120.0(8)
C(74)-C(75)-C(76)	122.6(6)
C(71)-C(76)-C(75)	119.3(4)
O(1S)#1-C(1S)-C(1S)#1	0(3)
O(1S)#1-C(1S)-C(2S)	122(5)
C(1S)#1-C(1S)-C(2S)	122(5)
O(1S)#1-C(1S)-O(2S)#1	30(2)

C(1S)#1-C(1S)-O(2S)#1	30(2)
C(2S)-C(1S)-O(2S)#1	152(3)
O(1S)#1-C(1S)-C(2S)#1	30(2)
C(1S)#1-C(1S)-C(2S)#1	30(2)
C(2S)-C(1S)-C(2S)#1	152(3)
O(2S)#1-C(1S)-C(2S)#1	0.0(10)
C(1S)-C(2S)-O(1S)#1	28(3)
C(1S)-C(2S)-C(1S)#1	28(3)
O(1S)#1-C(2S)-C(1S)#1	0.0(18)

Symmetry transformations used to generate equivalent atoms: #1 −x+1, −y+1,-z+2
